# l-Arginine as a Bio-Based Curing Agent for Epoxy Resins: Glass Transition Temperature, Rheology and Latency

**DOI:** 10.3390/polym14204331

**Published:** 2022-10-14

**Authors:** Florian Rothenhäusler, Holger Ruckdaeschel

**Affiliations:** 1 Department of Polymer Engineering, University of Bayreuth, Universitätsstraße 30, 95447 Bayreuth, Germany; 2 Neue Materialien Bayreuth GmbH, Gottlieb-Keim-Straße 60, 95448 Bayreuth, Germany

**Keywords:** l-arginine, sustainability, epoxy resin, DSC, viscosity, latency, amino acid

## Abstract

The need for sustainable practices in the processing chain of fiber-reinforced thermosets has led to the development of bio-based epoxy resins and curing agents. As a contribution to sustainable composites, this study focuses on the glass transition temperature ($T_{g}$), viscosity and latency of diglycidyl ether of bisphenol a (DGEBA) cured with l-arginine in the presence of a urea-based accelerator. These characteristics are decisive features for application as a matrix in fiber-reinforced polymer composites produced via prepreg technology in which low viscosity and sufficient latency, meaning low reactivity of the one-component system, are necessary. The homogeneous mixture of amino acid and epoxy resin was prepared via three-roll milling. Two formulations, Argopox-1 with 1 wt.% accelerator and Argopox-2.5 with 2.5 wt.% accelerator, were prepared and parts of each formulation were stored at 22 °C and −18 °C, respectively. Both formulations were tested via differential scanning calorimetry (DSC) and small amplitude oscillatory shear rheology (SAOS) after 0 d, 30 d, 60 d, 90 d and 180 d of storage to determine the influence of accelerator weight fraction, storage temperature and storage period on the glass transition temperature of the uncured resin system $T_{g0}$, and their viscosity. The $T_{g}$ of the thermosets is about 100 °C. The DSC and SAOS measurements show that the $T_{g0}$ of Argopox-1 shifts about 5 °C in 60 d, while its viscosity is still low enough to be processed in a prepreg production line. Furthermore, Argopox-1 is storable for at least 180 d at −18 °C without significant changes in its $T_{g0}$ and viscosity. Consequently, Argopox-1 possesses a sufficiently high $T_{g}$ and adequate latency, as well as a low viscosity for application as prepreg matrix material.

## 1. Introduction

Glass and carbon fiber reinforced epoxy matrix composites play an important role in industries such as sports, automotive, wind energy, and aerospace due to their high weight-specific strength and modulus [1]. Here, pre-impregnated fiber products, so-called prepregs, allow the precise adjustment of fiber volume content while shortening process cycle times. Epoxy resins are commonly-used prepreg matrix systems due to their storability, high latency and low viscosity during fiber impregnation [2]. However, many curing agents for epoxy resins, such as amines, anhydrides and phenolic compounds, show toxic or carcinogenic effects [3,4,5,6]. These compounds are usually derived via chemical processes from petroleum which consume a lot of energy, and thus release a lot of CO_2_ during production or incineration. Therefore, substituting petroleum-based compounds with bio-based alternatives results in lower CO_2_ emissions and thus contributes to climate protection [7].

**Figure 1:**
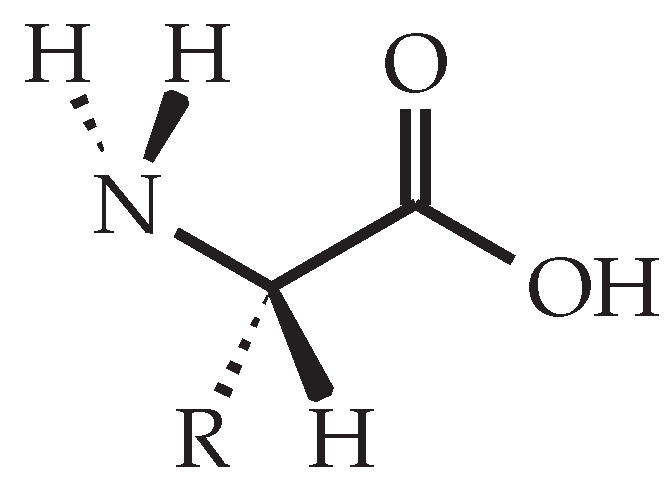
General structure of an l-amino acid that shows the characteristic amine (-NH_2_) and carboxylate (-COOH) functional groups as well as the side chain (-R).

In contrast to petroleum-based amine hardeners, such as diethylenetriamine or isophorone diamine, amino acids are non-toxic, bio-based and bio-degradable compounds [8,9]. Amino acids are characterized by their amine (-NH_2_) and carboxylate (-COOH) functional groups, as well as a side chain (-R) that is distinct for each amino acid (see Figure 1) [10]. As amino acids have the same amine groups as their petroleum-based counterparts, they are environmentally friendly alternatives for amine curing agents.

**Figure 2:**
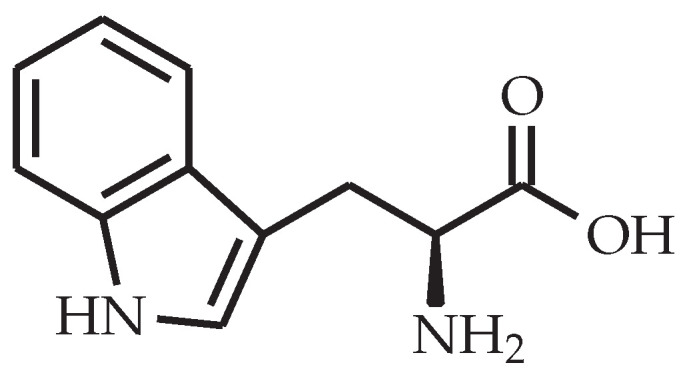
Chemical structure of l-tryptophan.

**Figure 3:**
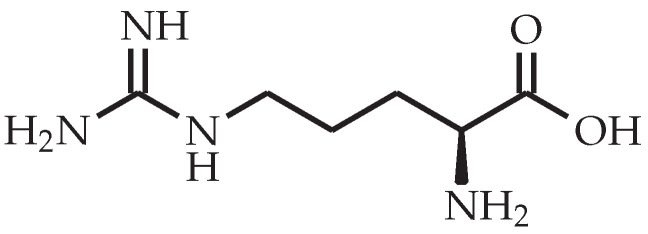
Chemical structure of l-arginine.

Previous investigations on the potential of amino acids as bio-based curing agents focused mainly on the reaction kinetics and glass transition temperature $T_{g}$ of petroleum-based epoxy resins cured with l-tryptophan [11,12,13,14]. Here, the $T_{g}$ of l-tryptophan cured DGEBA ranges from 66 °C to 101 °C if ureas were used as accelerator [13,14]. In other investigations, the reaction between l-tryptophan and DGEBA was accelerated by imidazoles which resulted in glass transition temperatures of 84 °C to 104 °C [11,13,15]. The range of $T_{g}$ from these investigations arises from the different ureas and imidazoles used as accelerators as well as the different ratios of epoxy resin to l-tryptophan. However, none of the previous investigations, except Shibata et al. [16], used a distinct stoichiometric ratio *R* of active hydrogen atoms from l-tryptophan to the epoxy groups of the resin.

l-tryptophan looks like a promising curing agent due to its aromatic structure that poses steric hindrance between cross-links, thus preventing rearrangement of network segments between cross-links which in turn results in a high $T_{g}$. However, the number of active hydrogen atoms in l-tryptophan is only four (see Figure 2), whereas other amino acids, such as l-glutamine, l-citrulline and l-arginine (see Figure 3), have more active hydrogen atoms. A higher number of amine groups in an aliphatic molecule decreases the free chain length in between them, thus leading to a higher number of cross-links with that curing agent as well as a higher $T_{g}$ [17].

Usually, amino acids come in the form of coarse powders [18]. Therefore, investigators usually grind the amino acids into a fine powder before mixing with epoxy resin, without specifying the method used or the resulting particle size distribution [11,19]. Other investigators used solvents, such as ethanol, methanol, acetone or aqueous acetic acid, to create a homogeneous mixture of epoxy resin and amino acid [15,16,20]. However, using solvents reduces the positive effect of the bio-based curing agent on the carbon footprint of the material. Furthermore, only small masses, usually smaller than 1 g, of curable mixture were attained via this method. Therefore, investigations on amino acids as curing agents focused mainly on experiments that do not require large sample sizes, such as differential scanning calorimetry, Fourier-transform infrared spectroscopy and thermo-gravimetric analysis.

However, important processing properties and requirements for the application of amino acids as bio-based curing agents, such as the viscosity and latency of the resin system, were not investigated. Here, latency refers to the compound’s ability to maintain its original properties, such as $T_{g0}$, viscosity and $T_{g}$ after curing, at set storage conditions for a prolonged storage period. Thus, chemical reactions between resin, curing agent and accelerator should be minimal at storage temperature. Latency is a prerequisite for the industrial application of thermosetting compounds as these must have a preferably long shelf-life at room temperature (T= 22 °C). For thermosetting resins, the storage period at room temperature in which the compound’s viscosity doubles due to pre-conversion is called the pot-life of the material system [21]. However, aging does not only affect the viscosity of the uncured resin system but also alters the processing properties of the prepreg, such as tack, and the matrix-driven mechanical properties of the cured laminate, such as tensile strength perpendicular to fiber direction and interlaminar shear strength [22,23].

Therefore, the objective of this investigation is to study the glass transition temperature, viscosity and latency of an amino acid-cured epoxy resin. The goal is to check whether the material system might be a suitable matrix material for fiber-reinforced polymers produced via prepreg technology. In this study, l-arginine is used as a curing agent due to its high number of active hydrogen atoms, as well as its low price and good availability. Furthermore, a solvent-free, more environmentally friendly approach to creating homogeneous amino acid epoxy resin mixtures via three-roll milling is presented.

## 2. Materials and Methods

### 2.1. Materials

D.E.R. 331 with an epoxide equivalent weight of 187 g mol^−1^ from Blue Cube Assets GmbH & Co. KG, Olin Epoxy (Stade, Germany) was used as the resin. l-arginine with a purity of 98.9% was bought from Buxtrade GmbH (Buxtehude, Germany). The reaction between amino acid and epoxy resin was accelerated by the substituted urea DYHARD^®^ UR500 by Alzchem Group AG (Trostberg, Germany).

### 2.2. Sample Preparation

Similar to the investigation by Shibata et al. [16], it was assumed that l-arginine has seven active hydrogen atoms. Therefore, the amine equivalent weight of l-arginine is approximately 24.9 g mol^−1^. A homogeneous mixture (stoichiometric ratio *R* = 1) of D.E.R. 331 and l-arginine was prepared using a dual asymmetric centrifuge Speed mixer by Hauschild Engineering (Hamm, Germany) at 3000 min^−1^ for 120 s.

To decrease the particle size of the amino acid, the mixture of DGEBA and l-arginine is processed using a three-roll mill type EXAKT 120 EH-450 by EXAKT Advanced Technologies GmbH (Norderstedt, Germany). This method has already been applied for dispersing different fillers in epoxy resin [24,25] and proved to be advantageous compared to solvent-based methods [26]. The temperature of the rolls was set to 25 °C. The gap between the feed and the middle roll was set to 25 μm, whereas the gap between the middle and apron roll was 5 μm. The number of revolutions of the apron roll was set to 250 min^−1^. The numbers of revolutions of the feed roll and middle roll were set automatically by the three-roll mill. Here, the ratios of the number of revolutions of feed roll to middle roll to apron roll are 1:3:9. The small gap combined with the high number of revolutions leads to shear rates in the gap in the order of magnitude of 10^5^ s^−1^. The stress exerted by the fluid on the particles results in a break-up of particles. The mixture was run through the three-roll mill five times to ensure sufficiently small particle sizes and a narrow particle size distribution.

Afterwards, two different formulations, one with 1 wt.% and one with 2.5 wt.% of accelerator were prepared (see Table 1). For the sake of simplicity, the formulations are referred to as Argopox-1 and Argopox-2.5. The accelerator was mixed into the resin with the dual asymmetric centrifuge speed mixer at 3000 min^−1^ for 120 s. Afterwards, the formulations were kept in an vacuum oven at 45 °C and 20 mbar for 1 h to ensure the elimination of entrapped air.

### 2.3. Sample Storage

One part of each formulation was stored at room temperature (T= 22 °C), while the other part was stored at T= −18 °C. The different formulations in each storage condition were tested after preparation (0 d) and after storage for 30 d, 60 d, 90 d. The formulations stored at T= −18 °C were also tested after 180 d.

### 2.4. Characterization Methods

Dynamic DSC measurements were carried out to determine the increase in the glass transition temperature of the uncured mixture $T_{g0}$ and the glass transition temperature of the cured compound $T_{g}$ in dependence of storage time. A Mettler Toledo DSC 1 (Columbus, OH, USA) was employed with a heating rate of 10 K min^−1^ from −50 °C to 275 °C. The flow of nitrogen was set to 50 mL min^−1^. The sample mass was 20 ± 2 mg. Three specimens were tested to ensure sufficient reproducibility.

The effect of aging on the viscosity increase in the uncured mixture was measured with an Anton Paar MCR 302 (Graz, Austria) plate-plate rheometer. The plate diameter was 25 mm and the gap between plates was set to 1 mm. A constant heating rate of 3 K min^−1^ from 25 °C to 180 °C was used. The shear amplitude was set to 5% with a shear frequency of 1 rad s.^−1^

The cured thermoset was characterized by a Zeiss Gemini 1530 Scanning Electron Microscope by Carl Zeiss AG (Oberkochen, Germany). The acceleration voltage used was 3 kV and the surfaces were platinum-sputtered with a thickness of about 5 nm.

## 3. Results and Discussion

### 3.1. Differential Scanning Calorimetry

**Figure 4:**
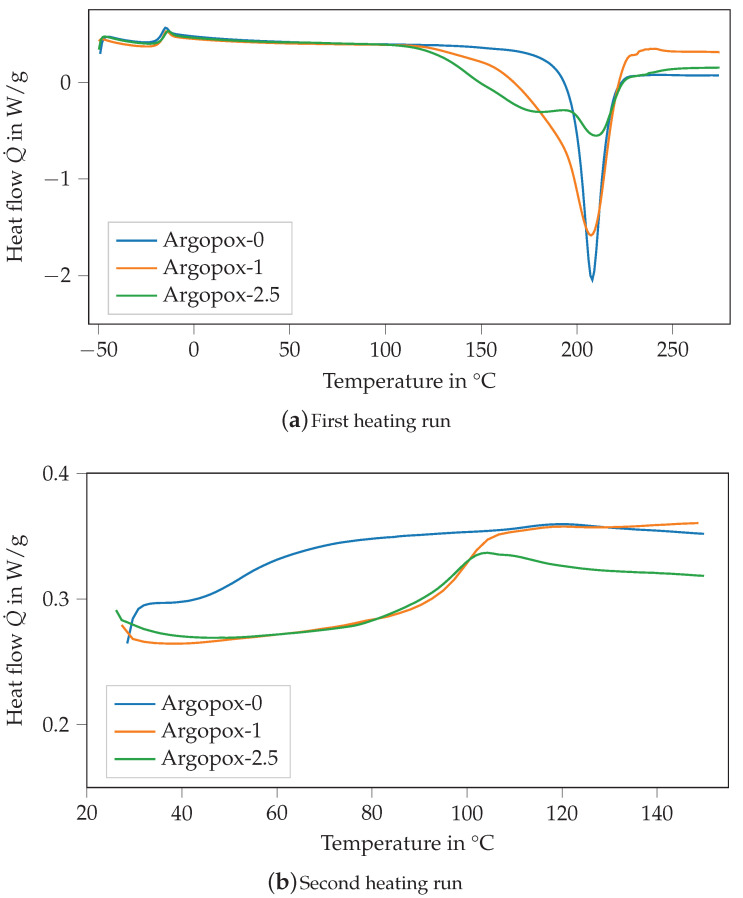
DSC thermograms of the mixture of DGEBA and l-arginine without any accelerator (Argopox-0, blue) as well as with 1 wt.% (orange) and 2.5 wt.% (green) of DYHARD^®^ UR500 of the first (**a**) and second (**b**) heating run.

Figure 4 shows the DSC thermograms of the mixture of DGEBA and l-arginine without any accelerator (Argopox-0, blue) as well as with 1 wt.% (orange) and 2.5 wt.% (green) of DYHARD^®^ UR500. With the addition of the accelerator, the exothermic peak of the heat flow becomes smaller but stays at around 208 °C (see Figure 4a). Additionally, the shape of the curve is altered so that a shoulder forms at around 180 °C. Thus, the start of the curing reaction is shifted to a lower temperature starting at 115 °C for Argopox-1 and 125 °C for Argopox-2.5 instead of 160 °C. The formation of the double peak shows that with the addition of more accelerators the activation energy for one step of the curing reaction is reduced significantly. Usually, the curing reaction between amines and epoxy resins takes place in two steps. Firstly, the primary amines react with epoxy groups to secondary amines. Secondly, the secondary amines react with epoxy groups to tertiary amines [27]. Additionally, l-arginine possesses several amine groups as well as an amine group that are all chemically different and therefore have different activation energies for the curing reaction. Still, the temperature $T_{\mathrm{peak}}$, at which the exothermic heat flow has its maximum, is considerably higher than that of DGEBA cured with dicyandiamide. Here, the maximum temperature for curing can be lowered down to less than 140 °C by the addition of suitable accelerators such as amines, imidazoles or ureas [28,29,30,31,32].

The $T_{g0}$ of the different mixtures is about −19 °C, whereas the $T_{g}$ of the cured systems is about 52.9 °C, 100.4 °C and 96.8 °C for Argopox-0, Argopox-1 and Argopox-2.5, respectively (see Figure 4b). The difference in $T_{g}$ can be explained by the difference in the heat of reaction of the formulations which are 193.7 J g^−1^, 339.2 J g^−1^ and 273.2 J g^−1^ for Argopox-0, Argopox-1 and Argopox-2.5, respectively. This shows that the addition of the accelerator facilitates the reaction between the amino acid and the epoxy resin. However, the addition of 2.5 wt.% of the accelerator lowers $T_{g}$ because the epoxy groups react with the accelerator rather than with l-arginine. The heat of reaction is comparable to that of DGEBA cured with dicyandiamide (350 J g^−1^ to 400 J g^−1^) [33]. Therefore, the heat of reaction in combination with the increase in $T_{g}$ during curing shows that three-roll milling is a suitable preparation method for a homogeneous mixture of DGEBA and l-arginine.

The $T_{g}$ of Argopox-1 and Argopox-2.5 are comparable to the glass transition temperatures of DGEBA cured with l-tryptophan [11,13,14,15]. Still, the $T_{g}$ of DGEBA cured with dicyandiamide is considerably higher at around 140 °C [22,28]. The difference in $T_{g}$ most likely stems from the aliphatic side chain of l-arginine which contains three carbon atoms between its α-amine group and the next functional group. The carbon chain is highly flexible which facilitates rearrangements of network segments, and thus lowers $T_{g}$.

**Figure 5:**
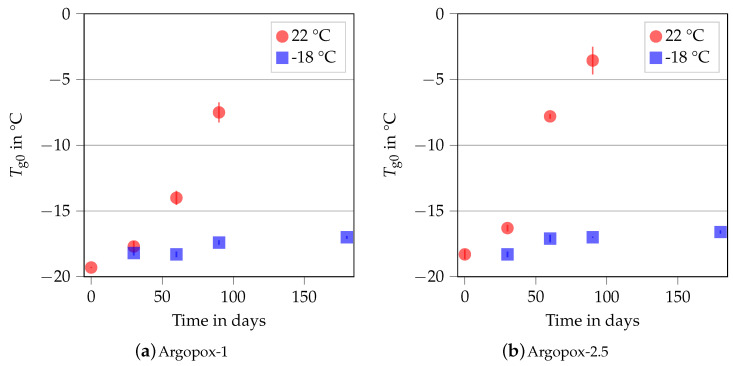
Glass transition temperature $T_{g0}$ of the uncured Argopox-1 (**a**) and Argopox-2.5 (**b**) in dependence of the storage period at T= 22 °C (red) and T= −18 °C (blue).

Figure 5 shows the $T_{g0}$ of Argopox-1 and Argopox-2.5 after different storage periods at 22 °C and −18 °C storage temperature. Here, $T_{g0}$ stays about the same at −18 °C storage temperature and is approximately −18 °C to −17 °C for both Argopox-1 and Argopox-2.5. However, the glass transition temperatures of the uncured systems increase steadily at room temperature. The $T_{g0}$ of Argopox-1 shifts from −19.3 °C to −14 °C after 60 d while the same shift in $T_{g0}$ of Argopox-2.5 is reached after a little more than a month. Hübner et al. found a shift of about 5 °C in $T_{g0}$ in an epoxy resin cured with dicyandiamide and accelerated with DYHARD^®^ UR500 as result of aging at room temperature after 91 d [22]. This shows that the l-arginine-cured epoxy resin is less latent, meaning more reactive, than the petroleum-based material system. For the epoxy cured with dicyandiamide, the increase in $T_{g0}$ is mainly attributed to the reaction between epoxy monomers and the accelerator [34]. The result of that reaction is DGEBA oligomers, whose increased molecular weight leads to an increase in $T_{g0}$ and viscosity. Despite the presence of l-arginine, it is likely that the same mechanisms are at work, meaning that the shift in $T_{g0}$ is attributed to the reaction between DGEBA and DYHARD^®^ UR500.

### 3.2. Viscosity

**Figure 6:**
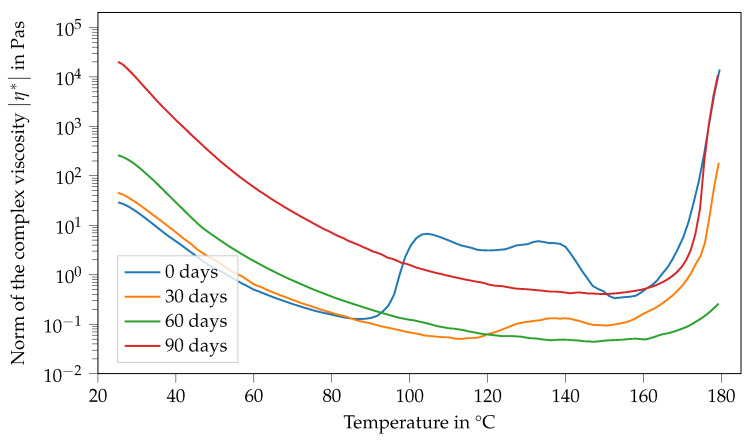
Norm of the complex viscosity of Argopox-1 between 25 °C and 180 °C after storage periods of 0 d, 30 d, 60 d and 90 d at T= 22 °C.

Figure 6 shows the viscosity of Argopox-1 between 25 °C and 180 °C after different storage periods. Here, the viscosity curve and its change with storage time show several characteristics. Firstly, the viscosity function in the fresh state, meaning the storage period is 0 d and decreases with increasing temperature up to 88 °C. Afterwards, viscosity increases rapidly from 0.1 Pa s to 6.7 Pa s. Then, there is a second peak at around 133 °C, followed by a sharp decrease down to 0.3 Pa s at 152 °C. Finally, the viscosity increases steadily up to 14,000 Pa s at 180 °C. After storage of one month at 22 °C, the viscosity function shows characteristics that are similar to the fresh state. However, there is only one viscosity peak which is also considerably smaller, reaching only 0.13 Pa s at 140 °C. Furthermore, the peak is shifted to higher temperatures showing that the mixture became less reactive during storage. Additionally, the viscosity at 25 °C increased from 29 Pa s to 45 Pa s within one month of storage. This trend holds true for the viscosity curve after two months of storage. Here, the viscosity at 25 °C increased to 260 Pa s.

As already mentioned above, the change in $T_{g0}$ is most likely mainly attributed to the reaction between DGEBA and the urea accelerator. Naturally, the increased molecular weight of the DGEBA oligomers leads to increased viscosity. Based on the hypothesis that the changes in $T_{g0}$ and viscosity at 25 °C are attributable to the accelerator, it is likely that the viscosity jump depends on the concentration of the accelerator. Interestingly after two months of storage, there is no peak in viscosity at higher temperatures visible. Moreover, the final viscosity increases due to curing occurring at higher temperatures, showing that the system becomes even less reactive over time. However, the material system is more reactive and cures at lower temperatures after three months of storage than after storage for one or two months, respectively. Here, the viscosity at 25 °C increases to approximately 20,000 Pa s.

**Figure 7:**
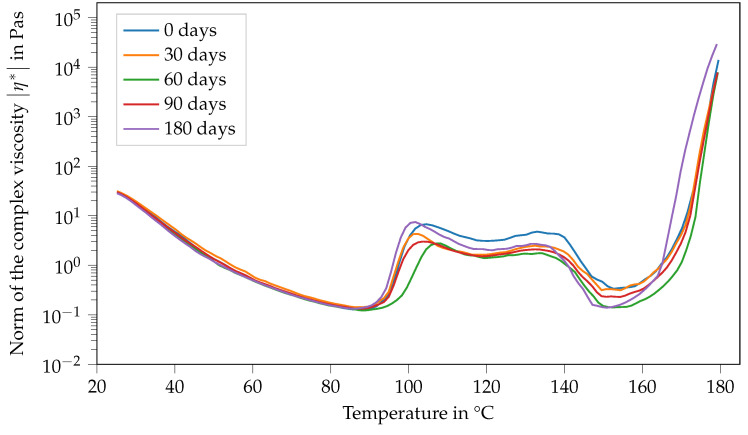
Norm of the complex viscosity of Argopox-1 between 25 °C and 180 °C after storage periods of 0 d, 30 d, 60 d, 90 d and 180 d at T= −18°C.

Contrary to that, the viscosity function of Argopox-1 shows no systematic trends regarding its change in dependence of storage time during storage at T= −18 °C (see Figure 7). Here, the same double peak in viscosity can be observed with slight variations regarding their values independent of the storage period.

**Figure 8:**
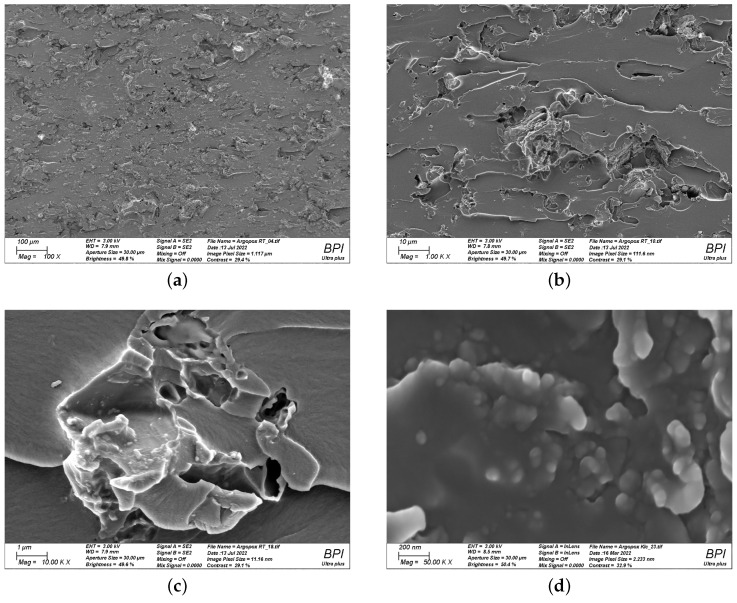
Scanning electron microscopy images of cured Argopox-1 at 100× (**a**); 1000× (**b**); 10,000× (**c**); and 50,000× magnification (**d**) showing the fracture surface of the cured thermoset and the nanometer-sized l-arginine particles.

To find a reasonable explanation for the observed double peak in the viscosity function of Argopox-1, the morphology of l-arginine in the epoxy resin has to be considered. The processing via three-roll milling leads to a decreased average particle size and a narrow particle size distribution. Figure 8 shows the fracture surface of cured Argopox-1 at 100×, 1000×, 10,000× and 50,000× magnification. Here, individual l-arginine particles with size between approximately 50 to 100 nm can be observed (see Figure 8d). This means that not all the amino acid is dissolved in the epoxy resin but that some of it is distributed homogeneously as fine particles in the matrix.

**Figure 9:**
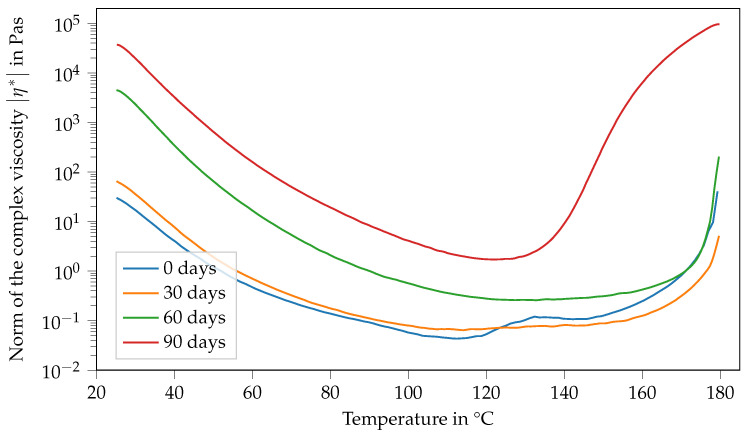
Norm of the complex viscosity of Argopox-2.5 between 25 °C and 180 °C after storage periods of 0 d, 30 d, 60 d and 90 d at T= 22 °C.

In general, the viscosity of a suspension depends, among other things, on the viscosity of the matrix, the particle volume fraction as well as particle aspect ratio. Another influencing factor might be the temperature-dependent solubility of l-arginine in DGEBA. As the formation of the double peak is different for Argopox-1 and Argopox-2.5 (see Figure 9), it is reasonable to assume that the cause for the viscosity increase is the concentration of DYHARD^®^ UR500. However, the exact mechanism behind the emergence of the double peak in viscosity is beyond the scope of this investigation and might be focused upon in the future.

Similarly to the results of Argopox-1, the viscosity of Argopox-2.5 shows a peak in its fresh state at around 132 °C (see Figure 9). However, the peak of Argopox-2.5 is considerably smaller than that of Argopox-1 and more comparable to the viscosity function of Argopox-1 after one month of storage at 22 °C. After one month of storage at 22 °C, Argopox-2.5 shows no peak in viscosity. The viscosity at 25 °C increases continuously with prolonged storage, from 30 Pa s in the fresh state to 37,000 Pa s after 90 d of storage at 22 °C. Similarly to the trends observed for Argopox-1, the reactivity of Argopox-2.5 increases again after a certain storage period so that the curing and thus the increase in viscosity starts at lower temperatures.

**Figure 10:**
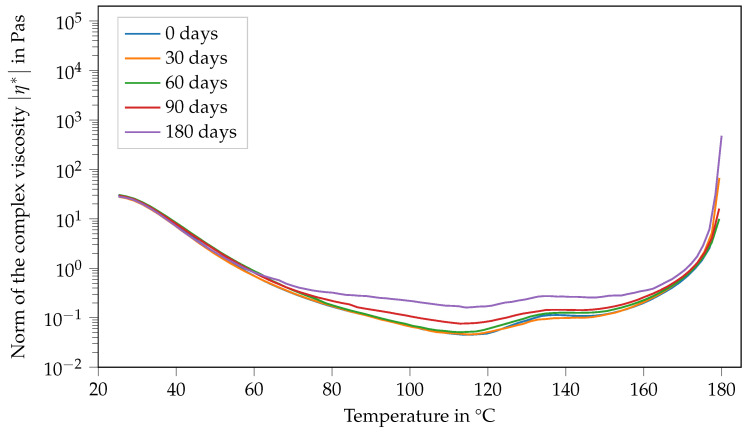
Norm of the complex viscosity of Argopox-2.5 between 25 °C and 180 °C after storage periods of 0 d, 30 d, 60 d, 90 d and 180 d at T= −18°C.

Interestingly, storage at −18 °C has a more systematic influence on the viscosity of Argopox-2.5 than on that of Argopox-1 (see Figure 10). Here, the peak in viscosity occurs at approximately the same temperature (132 °C). However, the resin mixture becomes more reactive, meaning that the viscosity increase starts at lower temperatures and the peak value of viscosity is higher with prolonged storage periods.

## 4. Conclusions

This study focused on the glass transition temperature, viscosity and latency of l-arginine cured DGEBA in the presence of a urea-based accelerator. A solvent-free processing method for the mixture of epoxy resin and amino acid via three-roll milling was presented and its suitability as a preparation method was confirmed via DSC. The $T_{g}$ of the cured epoxy resin is about 100 °C which is sufficient for thermosetting matrix systems for prepregs. However, the $T_{g}$ that results from the bio-based curing agent l-arginine is considerably lower than that of dicyandiamide due to the aliphatic side chain of l-arginine. The DSC and SAOS measurements show that the $T_{g0}$ of Argopox-1 shifts about 5 °C in 60 d, while its viscosity is still low enough to be processed in a prepreg production line. Here, the typical processing window of prepreg production lines ranges from 0.1 Pa s to 10 Pa s at temperatures between 25 °C and 80 °C. Furthermore, Argopox-1 is storable for at least 180 d at −18 °C without significant changes to its $T_{g0}$ and viscosity. It is concluded from the combined results of DSC and SAOS that the increase in $T_{g0}$ and viscosity are driven by the urea accelerator. As there are other urea-based accelerators available, it would be interesting to see their effect on the $T_{g}$ and latency of the resin system. The addition of flame-retardants or other additives requires a low matrix viscosity, which is given by Argopox-1 (0.1 Pa s). Therefore, the properties of Argopox-1 can be adjusted to the requirements of different applications. As Argopox-1 possesses a $T_{g}$ of about 100 °C and sufficient latency, as well as a low viscosity for prepreg production, the investigation of its mechanical properties is the next logical step to introduce amino acid cured epoxy resins as prepreg matrix materials.

## Figures and Tables

**Table 1:** Composition of Argopox-1 and Argopox-2.5.

Component	Argopox-1	Argopox-2.5
D.E.R. 331	87.4 wt.%	86.0 wt.%
l-arginine	11.6 wt.%	11.5 wt.%
DYHARD^®^ UR500	1 wt.%	2.5 wt.%
